# Two Clinically Implementable Digital PCR Assessments of DNA Methylation for Diagnosing Heavy Alcohol Consumption

**DOI:** 10.3390/epigenomes9010001

**Published:** 2024-12-24

**Authors:** Robert Philibert, Steven R. H. Beach, Allan M. Andersen

**Affiliations:** 1 Department of Psychiatry, University of Iowa, Iowa City, IA 52246, USA; allan-andersen@uiowa.edu; 2 Behavioral Diagnostics LLC, Coralville, IA 52241, USA; 3 Department of Psychology, University of Georgia, Athens, GA 30602, USA; srhbeach@uga.edu

**Keywords:** alcohol, alcohol dependence, seizures, DNA methylation, digital PCR, in vitro diagnostics

## Abstract

Background: Heavy alcohol consumption (HAC) has a profound adverse effect on human health. Unfortunately, there is a relative lack of tools that are easily implementable in clinical settings and that can be used to supplement self-reporting in the diagnosis and management of HAC. In part, this paucity is due to limitations of currently available biological measures and a mismatch between available biological measures and the needs of clinicians managing HAC. Objectives: We first review the pros and cons of existing biological measures. Next, we review the underlying theory and the performance characteristics of two recently developed methylation-sensitive digital PCR (MSdPCR) assays, referred to as the Alcohol T Score (ATS) and ZSCAN25, for the assessment of chronic and recent HAC, respectively. Finally, we outline a paradigm for improving the clinical diagnosis and management of alcohol use disorders by utilizing these new markers of alcohol consumption. Conclusions: We conclude that further studies to understand the test performance characteristics of each of these epigenetic tools in larger, diverse populations are in order.

## 1. Are the Harmful Effects of Heavy Alcohol Consumption Underestimated?

Heavy alcohol consumption is a significant medical concern. According to the United States Centers for Disease Control (CDC), alcohol consumption is the third leading preventable cause of death in the United States [1,2]. Annually, the costs of treating alcohol use disorders are estimated to exceed USD 100 billion [3]. Alarmingly, it is likely that this high figure underestimates the true impact of alcohol use on the health of the American public. Currently, the best estimates from the CDC are produced by the Alcohol-Related Disease Impact (ARDI) program [4], and ARDI estimates of alcohol-attributable death and alcohol-attributable fractions (AAF) of harms are calculated using estimates of the total proportion of deaths for various causes that are attributable to alcohol use [4] based on the current scientific literature. Unfortunately, for many of the alcohol-attributable conditions, the vast majority of the scientific literature concerning the impact of alcohol on medical outcomes is based on self-reports of the patients.

Relying on self-reported alcohol consumption is known to be problematic, especially for those with patterns of heavy use [5,6]. In 2021, Neilsen and colleagues reviewed the literature and found 11 studies that compared biomarker data to self-reports and found substantial differences between objective indicators and self-reported levels of alcohol consumption [6]. Yet, when calculating indirect AAFs, ARDI uses the self-reported alcohol use levels reported by those 20 years and older. This approach should be expected to lead to substantial underestimates. Indeed, when nationwide data collected between 1993 and 2006 were analyzed by Neilsen and associates, they found that the per capita estimates of alcohol sold were on average three to four times higher than self-reported per capita consumption [7]. As a result, there is considerable reason to believe that the estimates of both medical and economic impacts of alcohol are underestimates and that there is an urgent need for better tools to assess alcohol use patterns.

One potential source of innovative approaches for the assessment of HAC relies on advances in the use of DNA methylation to characterize alcohol use patterns. An inspiration for this approach is the striking success using DNA methylation techniques to assess another form of substance use, smoking status. With respect to smoking, measurements of cg05575921 methylation are now generally accepted as a reliable and useful method of establishing smoking status [8,9]. Still, progress in epigenetics has been slow, and it is critical to note that this consensus with respect to cg05575921 only occurred after hundreds of studies on the matter, including large numbers of case/control analyses. Furthermore, the pathway to consensus relied on the availability of existing, easily employed, Food and Drug Administration-approved biomarkers, such as cotinine and exhaled carbon monoxide, for confirming the presence or absence of smoking in study subjects. Unfortunately, there are no FDA-approved biomarkers for heavy alcohol consumption (HAC), and the number of high-quality DNA methylation analyses of alcohol use are more limited, with very few of the studies actually employing existing alcohol use biomarkers to confirm the alcohol use status.

In this communication, we review the extant literature of genome-wide methylation studies of alcohol use, then describe a methylation-sensitive digital PCR (MSdPCR) approach for quantifying alcohol consumption. We will identify gaps in the literature and suggest a framework for using methylation-based tools for characterizing alcohol use patterns in the research and clinical settings.

## 2. Current Status for the Determination of Heavy Alcohol Consumption

### 2.1. Self-Report

The most common method of quantifying heavy alcohol consumption for both clinical and research settings is self-report. To date, there is not extensive literature describing the biochemical validation of self-reports of alcohol in the research setting. For example, for the larger longitudinal biorepositories, such as the National Health and Nutrition Examination Survey (NHANES), no alcohol biomarker testing was performed to validate associations. In part, this lack of biochemical validation stems from the high costs of conducting biological testing, the reluctance of many in the field to question the self-reporting of their research subjects, and an absence of strong external forces to ensure rigor. In addition, at lower levels of alcohol use, and in non-medical settings, self-report may provide a useful and low-cost approach to characterizing individual differences in alcohol use. However, the problem of underreporting alcohol use is particularly apparent among heavy users [10], and this may attenuate correlations of HAC with social risk factors, as well as medical consequences. Underreporting alcohol may be particularly pronounced among binge drinkers [10,11], who are also of particular interest in medical contexts. Indeed, the broader problem of underreporting of stigmatized behavior has been very well illustrated for cigarette smoking, e.g., there was substantial underreporting of smoking in the Framingham study, with only 8% self-reporting smoking, although almost 50% showed some biological evidence of smoking [11]. Again, reliance on self-reporting in this context would suppress associations of smoking with concurrent chronic illness. We expected similar problems with self-reported HAC [12,13], and have shown that both alcohol use and smoking are substantially underreported [11] and that these measurement problems lead to non-significant associations of self-reported alcohol use and cigarette consumption with accelerated aging and cardiac risk [14,15,16,17,18].

Given the elevated stakes of accurate assessment of HAC in healthcare settings, and the problems with reliance on self-reporting, it is especially important that non-self-report alternatives be available to supplement reliance on self-reports. This is well recognized in many clinical contexts. For example, in order to receive a liver transplant, patients must undergo extensive testing to ensure that they are not surreptitiously drinking [19]. In both inpatient and substance use treatment, biochemical validation of alcohol use status is routinely employed. For example, after holidays or weekends, outpatients in alcohol treatment programs are often tested using breathalyzers or saliva testing to ensure that relapse has not occurred. Life insurance underwriters routinely request biological testing before agreeing to accept policy applications from potential clients. Finally, because of the potential legal ramifications for both the failure to diagnose an alcohol use disorder or potential alcohol withdrawal syndrome (AWS), clinicians in emergency rooms and in patient alcohol treatment facilities routinely request biochemical assessment of alcohol status. As we note below, the limitations of available assessments suggest the need for additional methods.

### 2.2. Breathalyzer

In acute settings, clinicians rely on “breathalyzer” assessments that measure the concentration of alcohol in exhaled air [20]. While extremely useful for determining current intoxication, these assessments are not informative about longer-term consumption patterns. Instead, for the assessments of more sustained consumption, clinicians rely on algorithms that incorporate measured blood levels of liver proteins (e.g., alanine aminotransferase (ALT), γ-glutamyl transferase (GGT), aspartate aminotransferase (AST), or carbohydrate-deficient transferrin (CDT)) or metabolites, such as ethyl glucuronide (EtG) [21,22].

### 2.3. Liver Function Tests (LFTs)

Generations of clinicians have used LFTs as their “go to” method for assessing chronic alcohol use. In part, this reliance stems from the traditional utility of these assessments in the general routine physical examinations of patients. For example, increased ALT levels are indicative of hepatitis [23]. When used with respect to determining alcohol use status, when present, elevated AST/ALT ratios are strongly indicative of alcohol-induced liver damage [24]. However, overall, the use of liver enzymes is regarded too insensitive and non-specific for quantifying alcohol consumption [25]. Furthermore, meta-analyses show only a modest ability of LFTs for predicting potential important clinical consequences of chronic alcohol use, such as AWS [26]. Therefore, clinicians are turning to more specific “purpose-built” tests for assessing alcohol use patterns.

### 2.4. CDT

For the past thirty years, probably the most commonly used serological test that is specifically used for assessing chronic alcohol consumption is the CDT [27]. First described by Stibler and associates in 1978, CDT testing assesses the quantity of sialic acid residues that are normally added to transferrin as part of post-translational processing [28]. The level of sialyation of transferrin, a key iron transport protein, can be decreased by drinking >80 g/day of alcohol for 2 to 3 weeks [24]. However, despite the promise of earlier studies, more recent studies have concluded that the overall sensitivity of CDT assessments for moderate current AUD is only 60%, with markedly lower sensitivity and specificity for milder forms of AUD [24,29]. Still, overall, CDT testing is regarded by many as the most accurate serum marker for chronic alcohol use [21,30]. Yet, in the clinical setting, CDT testing has increasingly been noted to have limitations. For example, for those clinicians interested in determining whether a patient needs to be hospitalized acutely for alcohol treatment, studies by two groups have shown that CDT has only modest power of the assay to predict severe AWS [29,31]. Furthermore, since the development of the test, the use of statins for the primary and secondary treatment of coronary heart disease has become common. Unfortunately, statins increase the global sialyation of serum proteins and, as a consequence, false-negative CDT results have been reported in those prescribed statins [32].

### 2.5. PEth

Phosphatidyl ethanolamine (PEth) may actually be better than CDT for detecting recent HAC [33]. PEth is a family of alcohol-specific phospholipid isoforms formed from phosphatidylcholine (PC) [34]. The exact frequency of each isoform is dependent on genotype, with the PEth species having an average half-life of about four days [34,35]. However, there is still significant debate as to which PEth isoforms are most optimal for quantifying alcohol consumption. Furthermore, PEth testing is typically performed using mass spectroscopy. Because relatively few healthcare facilities have these devices, and PEth levels of phlebotomy samples change as a function of time and temperature, the shipping and storage requirements of samples for PEth testing can present further challenges for clinical implementation [34]. Finally, with respect to potential for AWS, Novak and colleagues found that PEth levels did not predict severe AWS [36].

### 2.6. EtG

The final commonly used biochemical marker of alcohol consumption is EtG. EtG is a metabolite of alcohol whose accretion and excretion largely parallels that of alcohol, with its area under the curve (AUC) being absolutely proportional to that of ethanol itself [37]. EtG can be detected in the urine for several days after heavy ingestion of alcohol [38]. However, it does not detect alcohol use outside of that window and, as such, is not used for quantifying chronic use, nor is it useful for predicting AWS. Still, because the laboratory methods for assessing EtG status are relatively simple, EtG assessments have found a niche in less resource-intense care environments.

In summary, four types of biomarkers of alcohol intake: liver enzymes, CDT, PEth, and EtG, have been used to assess the history of alcohol consumption and potential of AWS. In the right settings, each of these tests can be extremely useful. However, at the current time, limitations in the clinical utility or ease of performance have sharply limited their use in both clinical and research settings. This situation has led to the need for alternative methods to biologically verify both recent and longer-term HAC.

## 3. The White Blood Cell as a Biosensor for Alcohol

One of the commonalities of the previously mentioned laboratory methods for detecting alcohol use is that to one extent or another, each of them rely on the liver [21,24]. The vast majority of serum AST, LDH, and AST levels originate from liver synthesis [21]. Similarly, transferrin is both synthesized and post-translationally modified in the liver [27]. Glucuronidation of alcohol mainly occurs in the liver [39]. Finally, while the pathway for synthesis of PEth is complex and can occur throughout the soma, the preferential sites for the majority of PEth found in mammals are in the muscle and liver [40]. Therefore, current biochemical methods lean heavily on the response of the liver for their analytical validity.

However, under many circumstances, the assumption of an unbiased response of the liver to alcohol intake is violated. For example, as previously noted, drugs such as statins alter the degree of sialylation of hepatically derived proteins, such as transferrin [32,41]. Furthermore, some high-prevalence diseases, such as hepatitis B and C, can make serum enzyme levels difficult to interpret [42]. Therefore, biological assessments whose outcomes are not heavily dependent on hepatic function could offer new windows for assessing alcohol consumption while side-stepping some of the potential limitations of current methods.

In this regard, epigenetic assessments of white blood cells (WBCs) may be a very useful alternative method for assessing alcohol consumption. Although their optimal function is dependent on a healthy liver, WBCs are part of the hematopoietic system, and in adults are produced in the bone marrow [43]. Unlike their red blood cell counterparts, their nucleus is both present and responsive to changes in the environment. For example, as is well documented through previous studies on smoking, DNA in the aryl hydrocarbon receptor repressor is demethylated and then actively transcribed in response to sustained exposure to the polyaromatic hydrocarbons found in tobacco smoke [44,45]. Therefore, there is considerable reason to believe that WBCs could serve as a biosensor, providing source material for epigenetic assessments of alcohol consumption.

The potential basis for WBCs to serve as indicators of alcohol consumption has been evident for many years. For example, the association of alcohol with alterations in WBC production has been known for almost a century, and it is well known in the medical community that chronic HAC is associated with impaired innate immunity [46,47,48]. Similarly, studies from the last century showed that chronic alcohol intake affects both the mobility and enzymatic activity of WBCs [49,50]. Therefore, it seems logical that examination of the relationship of changes in DNA methylation to heavy alcohol consumption could bear fruit due to the role of methylation as a key regulator of cell fate and function.

## 4. Promise and Challenges in Prior Epigenetic Studies of Alcohol Consumption

Based on that premise, over the past 15 years, a large number of studies have examined the relationship of alcohol use and DNA methylation. A PubMed literature search conducted in April of 2024 using the terms “alcohol”, “DNA methylation”, and “genome wide” identified 216 studies. The vast majority of these studies utilized WBC DNA from samples of convenience, such as large longitudinal studies, which had self-report data for alcohol consumption (e.g., drinks per day) but did not conduct analyses with respect to diagnostic classification schemes. In 2022, Dragic and colleagues conducted a systematic review of that literature and identified 11 cross-sectional studies ranging in size from 88 to 9643 subjects, and concluded that “potential methylation markers had been identified, but that further validation was needed” [51].

However, there are also a number of studies, such as Zhang and colleagues and ourselves, who used populations enriched for “case” subjects who had been medically ascertained to be heavy drinkers [52,53,54]. In 2021, Longley and colleagues conducted a systematic review of 27 studies that focused on those with AUD, including a small number of studies that examined brain tissue itself [55]. In contrast to the weaker findings of Dragic and colleagues, Longley and colleagues concluded that “184 genes and 15 gene ontological pathways were differentially methylated in at least two studies” [55]. Unfortunately, although helpful in identifying lapses in the literature and opportunities for improvement, neither review conducted a true meta-analysis of the methylation data themselves. Nonetheless, jointly, these two well-written reviews indicate that the epigenomic response to alcohol intake is complex, and that many, many regions of the epigenome are affected.

Dealing with heterogeneity in studies. The findings by Longley and colleagues, which focused on those with AUD, were also complicated by at least two sources of heterogeneity: first, the type of DNA specimen, and second, the method of proband classification (e.g., type of AUD or alcohol consumption pattern) included in the studies. Most studies cited by Longley and colleagues used white blood cell DNA, but some analyses used brain or blood and brain DNA together. Since the DNA methylation signature is the hallmark of a cell’s identity and the relative response of a cell epigenome to a given exposure can vary as a function of cell type, some of the lack of consistency could be secondary to problems related to cell heterogeneity.

However, variation in the method of case control classification and the rigor used in assessing subjects is likely also a contributing factor. For example, given the difficulties in collecting brain samples post-mortem from subjects, it is easy to realize that full-length clinical interviews of subjects who contributed brains’ clinical status may not be available. However, a likely greater source of variation may be in the alcohol consumption patterns of subjects. The AUD “case” subjects included in the Longley meta-analysis included those admitted for acute intoxication as well as those who, although previously diagnosed with an AUD, may have not been drinking recently. Hence, with respect to the cadre of epigenetic studies of those with AUD, there is a great deal of heterogeneity with respect to type of DNA and the current clinical alcohol use patterns.

Understanding the effects of heterogeneity of case definition or DNA source on study results is essential to constructing clinical tools for clinicians. At the time these studies were conducted, it was not understood that the alcohol-induced methylation changes could revert as a function of abstinence. During the initial development of the literature in the field of substance use, this was not obvious, and often researchers would lump abstinent and current heavy alcohol users together as one group.

Thankfully, the insight into the potential differences between “state and trait” was provided by an unexpected source. During the course of reviewing one of our manuscripts, a reviewer suggested that the problem that we were encountering in understanding the epigenetic signal might be that the clinical diagnosis or “mindset” of the subject was irrelevant—it was only the recent exposure to smoke that was important. This insight directly led to use active, biochemically verified smokers and verified clean controls in our case/control analyses and the subsequent discovery of cg05575921 and the aryl hydrocarbon receptor repressor, which is now generally accepted as a biomarker for smoking status and intensity. To paraphrase the reviewer, “it is not what you think, it is what you do” that alters DNA methylation.

Applying the same line of thought to the review by Longley and colleagues, it is easier to understand the potential biases that may have contributed to the heterogeneity of the findings. Subtle variations in the method of clinic ascertainment or case definition can potentially result in marked changes in study results.

Applying these insights to our own processes has led to improvements in how we conceptualize AUD and alcohol consumption. For example, understanding that the type of DNA is critical to DNA methylation studies, we abandoned the use of lymphoblasts and have exclusively focused on whole blood DNA samples. The reason for abandoning lymphocytes is although they retain some of the signature of their donors, the methylation signature is markedly altered by Epstein Barr immortalization and cell culture conditions. This is regrettable because many, if not most, of the DNA collections at the Rutger’s Repository are in the form of lymphoblast cell lines. Second, because of their reliance on self-report, we have discontinued the use of DSM-based categories as the primary classification categories and have instead classified subjects on the quantity of alcohol consumed, such as the “heavy alcohol consumers” noted in our 2014, 2018, and 2019 studies. When possible, we have included only those subjects medically ascertained for the use of alcohol as case subjects and have used additional biomarkers of alcohol consumption, such as breathalyzer and carbohydrate-deficient transferrin levels, to provide further objective evidence of the amount of alcohol consumed. Even so, we have found unexpected heterogeneity in the type of heavy alcohol consumer that has been ascertained.

## 5. Why Use MSdPCR for Assessing Methylation Status?

Before any DNA methylation test can be clinically implemented, it is vitally necessary that it conform to the needs and expectations of the healthcare environment. First, to be maximally useful, the test should be capable of being performed rapidly. Indeed, part of the allure of the breathalyzer and, to a lesser extent, EtG analyses, is the rapid return of clinical information. Second, the test should be sufficiently accurate or informative. It should answer the clinical question being asked without the need for additional testing. Third, unsurprisingly, the test should also be affordable. The provision of healthcare is under increasing economic strain. Tests that inflate costs without delivering value do not gain market traction.

In contrast to most, if not all, existing methods, MSdPCR assessments of alcohol consumption successfully address each of these requirements. Table 1 provides a comparison of the four major methods of assessing DNA methylation currently used for in vitro diagnostics purposes. The use of methylation arrays and bisulfite sequencing techniques is limited by both reagent and instrumentation costs, as well as the time needed to conduct the assessments. Furthermore, while capable of delivering global assessments, at the individual locus level, array-based methods are the least accurate methods.

In contrast, both methylation-sensitive quantitative PCR (MSqPCR) and MSdPCR can be quickly and inexpensively performed. Because dPCR methods use later-generation PCR machines whose reagents can be a little more costly, dPCR-based methods have slightly higher consumable costs [56]. However, at scale, and in comparison to the rest of the laboratory costs, differences between the two PCR approaches are negligible and perhaps completely negated by the need for additional standards when performing qPCR under clinical conditions. From the operational standpoint, the principal differences between the two techniques are in the level of precision and the freedom of the measurement from an outside standard (see Table 1). Digital PCR is a reference-free technique whose level of precision can be easily quantified at scale, whereas quantitative PCR is a reference-dependent technique whose level of precision must be experimentally determined on an individual basis [57].

At the current time, most basic science researchers are not familiar with the use of MSdPCR to assess DNA methylation status. This is unfortunate, because this method is increasingly being used to for in vitro diagnostic purposes [58,59]. Like bisulfite sequencing and unlike both qPCR- and array-based methods, MSdPCR is a reference-free technique, which allows easy direct comparisons of results. For example, in order to determine the copy number of mRNA using qPCR, one must compare the rate of reaction in one tube to the rate of reaction in one or more other “reference” tubes containing a known concentration of the mRNA target. Conceptually, there are at least two sources of error in this approach. Errors in assessing the rate of reactions in the reference and “test” wells, as well as errors in the concentration of the mRNA in the “reference” well(s) [57]. In contrast, dPCR techniques first partition the “test” solution into thousands of discrete “droplets” or microwells. Then, after amplification is complete, they assess the presence or absence of a completed PCR reaction in each of the partitions. Finally, a Poisson distribution statistic is used to calculate the absolute concentration of the measurand [57].

**Figure 1:**
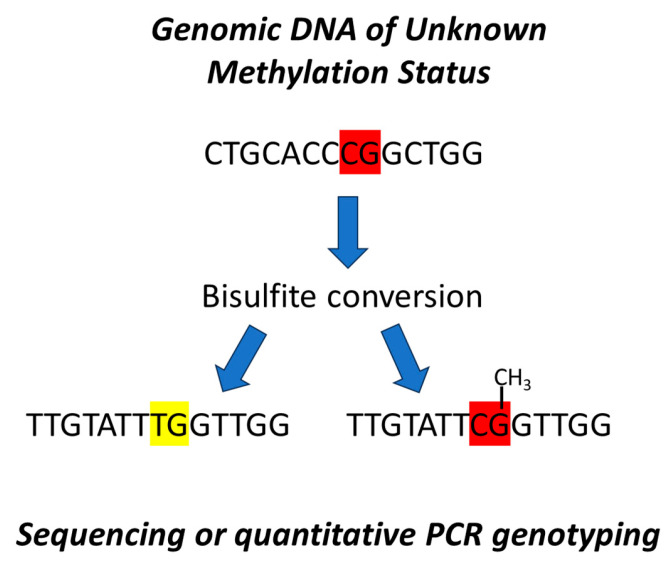
Determining cg05575921 methylation. As a first step, genomic DNA is treated with sodium bisulfite, which converts unmethylated cytosines to thymines while leaving methylated cytosine residues intact. The number of DNA segments containing either a C or a T residue at the original CpG site then can be determined using sequencing or PCR genotyping techniques.

Additionally, the dPCR approach has been modified to measure methylation ratios. In contrast to the original implementation of dPCR, which uses one reporter dye, MSdPCR uses two hydrolysable fluorescent probes, one specific for the methylated or “C” version of the original CpG site and one specific for the “T” allele representing the unmethylated version of the CpG site. By assessing the absolute concentration of both the “C” and “T” alleles, the percent of methylation and a 95% confidence interval of the measurement can easily be determined (see Figure 1).

Like most experimental techniques, a certain amount of planning is necessary to achieve optimal results. For example, when using sequencing or MSdPCR, the precision of the methylation ratio estimate is a function of the number of informative observations/droplets/wells. If the library copy number (i.e., number of independent copies of DNA) in the original sample is high (e.g., >1000), an adequate number of informative events are observed (e.g., >3000), there is no bias in any pre-amplification step, and the fluorescent probes are specific, either method is a robust, precise method for determining the methylation status of select CpG sites.

There are several different implementations of MSdPCR that have been described. The Bio-Rad system uses a microfluidic process to partition a 22 μL reaction volume into thousands of ~1 nanoliter aqueous droplets encapsulated in oil in each well of a standard 96-well plate. In contrast, the ThermoFisher AbsoluteQ and Qiagen Verati systems use pressure to force ~10 µL reaction volumes through microscopic fluid channels into grids of ~500 picoliter wells on specially designed flat chips containing up to 25,000 individual compartments. In either case, after partitioning, the fluid inside the droplets or chips is subjected to 30–40 rounds of thermocycling to complete PCR amplification of the CpG-site-containing amplicon and any requisite fluorescent probe hydrolysis. Then, each well or fluid droplet is interrogated with a laser to determine whether fluorescence corresponding to liberated “Fam” or “Hex” fluors is present. Using the Bio-Rad dPCR system, each of the fourteen MSdPCR assays currently being used or in the process of being clinically implemented have precisions of ~1% [45,58,60,61]. However, because each of the above dPCR systems are capable of assessing more than 15,000 droplets or wells in a single reaction, in actual practice the precision of each of the systems is fairly similar.

**Figure 2:**
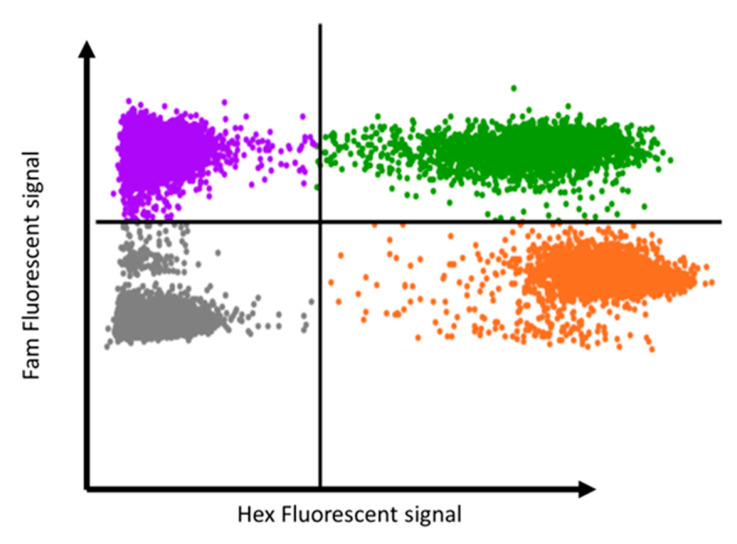
A typical 2D electropherogram output from methylation assessment using the AbsoluteQ digital PCR system. The purple, green, orange and gray dots represent wells with only C amplicons, C and T amplicons, only T amplicons or no amplicons, respectively.

There are several technical limitations in the use of MSdPCR to measure DNA methylation. First, when using a single volume partition approach, there are fixed constraints on precision, and achieving a 95% confidence interval of less than 0.5% requires the assessments of hundreds of thousands of partitions [62]. Second, not all CpG sites whose methylation can be quantified by sequencing can be modeled using PCR. For example, if one or more polymorphic SNPs are immediately adjacent to the CpG site, it may be difficult to design a probe that is specific for the given allele. Similarly, if the CpG site is in a low GC content region, the low melting temperature of the resulting bisulfite-converted DNA segment may also pose barriers to probe design. Still, we have found that by judicious design of the PCR primers and probes specific for the C and T alleles of the target site, excellent separation of the two fluorescent allele products for most variable CpG sites can be performed. For example, Figure 2 illustrates the 2D electropherogram of one such sample using the AbsoluteQ dPCR system by ThermoFisher. As the figure demonstrates, fluorescent Fam and Hex signals from the wells neatly segregate into clusters representing “C”, “T”, “C + T”, or null allele outcomes. Perhaps equally critically, the signal clusters in such a fashion as to allow “1D” calling of the allele signal, a property that makes the MSdPCR process easily compatible with high-throughput assessment processes.

Like all assessments of DNA methylation, the potential for genetic confounding must be addressed. As our studies to generate epigenetic tools for predicting incident heart disease demonstrate, the vast majority of the set points of the variable portion of the WBC methylome are modulated by cis and trans genetic variation [60]. Therefore, great care must be taken to identify sites that are minimally affected. Furthermore, because genetic variation can be ethnically specific, validation must be examined in diverse populations.

Finally, the speed and affordability of MSdPCR makes the approach highly scalable, which can provide more granular understanding of DNA methylation at select sites. To date, perhaps the best understood site using MSdPCR is that of cg05575921. We have determined DNA methylation at this site in over 10,000 samples using MSdPCR. What those studies have shown is that in both adolescents and adults who do not smoke, DNA methylation is approximately 86.2 ± 2.6%, with a non-linear dose-dependent demethylation in response to smoking. Critically, because the precision of MSdPCR is approximately 1%, it is clear that an essentially normally distributed natural variation of methylation in non-smokers occurs. However, when measured by reference-dependent array-based methods, the Beta value (or fractional methylation) for non-smokers can vary widely, in part as a result of error introduced by the normalization of the array signal. As a result, when using array-based measurements, average non-smoker values as high as 90% and as low as 80% have been observed in longitudinal studies [63,64,65].

## 6. Designing a Test Specific for Chronic Heavy Alcohol Consumption

The purpose of the proceeding discussions is to show how steady progress in both clinical and lab-analytical domains have set the stage for the use of MSdPCR assessments of methylation for characterizing alcohol consumption patterns. In essence, from the purely technological side, MSdPCR is a method that is capable of quickly, cheaply, and precisely assessing methylation values using WBC DNA. That still leaves the matter of identifying loci for MSdPCR assessment.

To identify CpG sites whose methylation is predictive of HAC, the first step that had to be settled was the clinical phenotype of alcohol consumption for the case and control analyses in which the algorithm would be developed and tested. We chose to focus on current chronic HAC, which we defined in 2014 as drinking an average of six or more drinks per day for the past eight weeks, because of the risk of this type of alcohol consumption pattern for important medical outcomes and its alignment with our laboratory understanding of how stable epigenetic signatures of environmental signatures evolve [66]. However, arriving at a case definition was not the only clinical issue that needed to be addressed. Because most heavy drinkers smoke, the potential overlap or confounding of the epigenetic signature of HAC with smoking still had to be addressed. Since up to 1/3 of the variable methylome can be affected by smoking, this is not a trivial task [67]. Therefore, in our 2018 genome-wide analyses of alcohol-associated DNA methylation, we specifically conducted analyses of a subset of HAC case and abstinent control non-smoking subjects, whose medical records, serological testing, and epigenetic testing confirmed their self-reports (see Table 3 from Philibert et al., 2018) [68]. By selecting those loci that were significantly differentiated in both the smoking and non-smoking subjects, but not significantly differentially methylated in smokers who did not drink, we could identify a set of markers specific to HAC itself.

In 2019, using this information and classification approach, we conducted a series of stepwise regression modeling analyses, first using the genome-wide data, then the MSdPCR data from 313 subjects (143 chronic HAC cases and 200 abstinent controls), to identify a four-marker panel capable of predicting HAC [69]. Not all loci nor all clinical subjects could be included in our analyses. First, because we intended to translate each of the array assessments into MSdPCR assays, all loci forwarded for possible clinical translation needed to have an arithmetic difference of methylation (i.e., ∆β) of greater than 4% to ensure that the effect size of HAC was markedly greater than the precision of the measurement. Second, in order to eliminate strong GxMethylation effects that could confound assessments, the distribution of β values at these loci was evaluated in nearly 700 genome-wide methylation arrays from abstinent adult controls and adolescents. Third, and finally, ensuring rigor in the assessments of the cases and controls was essential. All the HAC cases were recruited from inpatient alcohol treatment centers with their clinical records, including laboratory testing. These were reviewed to confirm their self-report data. The controls were selected from university-affiliated staff, with all self-report data for both cases and controls confirmed by biological testing. Only those cases and controls that met our clinical criteria were included in the final analyses.

Table 2 lists the four CpG sites that were identified and successfully translated into the ATS panel. From a theoretical standpoint, each of the sites have strong biological face validity. Cg02583484 is found in a cis-regulatory element (CRE) in HNRNPA1, a RNA-binding “hub” protein whose expression is strongly affected by alcohol [70]. Cg04987734 maps to a CRE in CDC42BPB, as serine/threonine kinase that is a regulator of the key cell cycle gene CDC42 that is critical for cytoskeletal organization and cell migration [71]. Cg09935388 maps to a CRE in GFI1, a zinc finger protein that is a critical regulator of hematopoiesis and, in particular, neutrophil production [72]. Finally, cg04583842 maps to a CRE in BANP, a direct negative regulator of p53 transcription [73]. Since defects in each of these processes can be linked to clinical phenomenology associated with alcoholism, such as neutropenia, altered immune function, and cancer, at face value, at a “70,000-foot level”, their association with methylation changes in our studies of HAC is not surprising. Still, the reason that they were selected is that the methylation status at each site independently predicts HAC status well.

Interestingly, principal component analysis shows that methylation at these sites loads on the same two principal components previously described for the methylomic response to alcohol [68]. In essence, all four of these assays are all capturing the same biological diathesis, with the improvement in prediction from their stepwise addition to the model resulting from the reduction in noise afforded by the effects of averaging the repeated measurements. Taking this step was necessary for gaining predictive reliability because, as opposed to changes at the smoking marker, cg05575921, where the ∆β is ~20%, the magnitude of the average difference between the cases and controls is only in the order of 5–8%.

Unfortunately, the “lumping” of the results of several loci together precludes the use of percent of methylation to describe the differences between the cases and controls. Therefore, to express this averaging process of the signal into an easily understood clinical metric, we converted each of the individual methylation assessments into Z scores (see the equation below). The ATS is simply the sum of the four Z scores, with the caveat that because two of the loci hypermethylated while the other two demethylated in response to alcohol, the signs of the two demethylating Z scores are changed so that a methylation change in the direction associated with alcohol use is always positive. The result of the manipulations is an easily understandable metric for expressing the steady-state rate of alcohol, with higher Z scores inferring steadily greater levels of alcohol consumption.

$$Z=\frac{M\;observed-Mcontrol\;avg}{\;Control\;SD}$$

where *M* = methylation and *SD* standard deviation.

**Figure 3:**
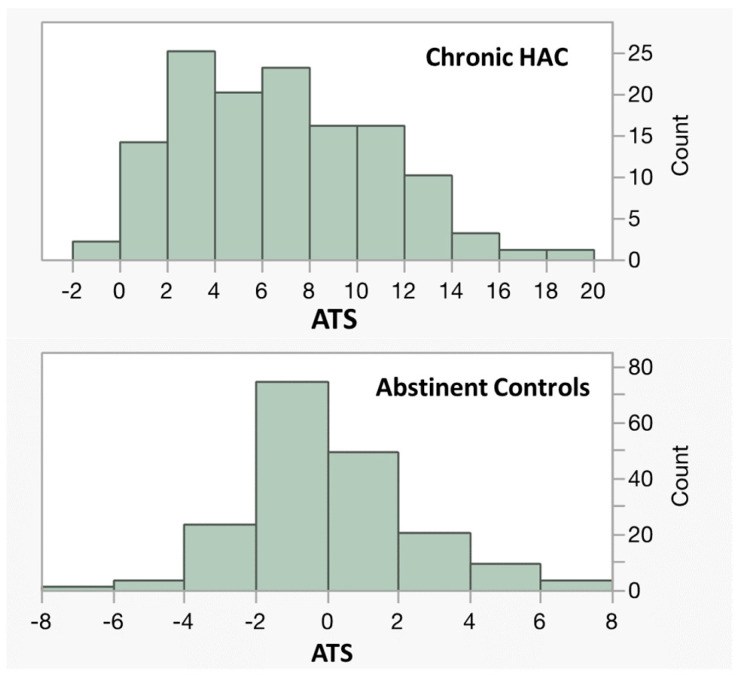
ATS values for inpatient subjects who reported chronic HAC (*n* = 131) and abstinent controls (*n* = 182).

Figure 3 illustrates the power of this method to distinguish chronic HAC subjects from controls in our 2020 study, for whom CDT values were available. The ATS values for abstinent individuals are zero-ordered with a standard deviation of 2.2. In response to sustained HAC, the Z score increases. The average Z score of the 131 chronic HAC subjects is 6.7, with a marked left skewing in the distribution. In these subjects, the CDT is strongly correlated with the ATS (r = 0.4, *p* < 0.0001) in the cases, but not in the controls (r = 0.01, *p* < 0.29). The overall area under the curve (AUC) for the ATS in differentiating cases from controls in this study was 0.96, with no evidence of sex bias. In contrast, the AUC for the CDT was only 0.87, with a marked gender bias.

Still, it is important to note that 35 of the subjects who reported chronic HAC and whose medical records strongly supported this history had ATS values of less than 3.5 (the high probability cutoff for HAC), while 80 HAC subjects had CDT levels of less than 1.6%, which was the testing laboratory’s cutoff for HAC for the CDT. The outliers on the biological tests suggest that either some of the subjects may have erroneously self-reported chronic HAC or that some heavy users are protected against elevated ATS or CDT by unknown factors. Since the average CDT of those with ATS < 3.5 was 1.33 ± 1.30% (*n* = 35), while the average CDT of those with an ATS > 3.5 was 3.14 ± 3.67% (*n* = 96), it is likely that those not predicted correctly using the ATS were lighter drinkers than those who were correctly predicted. Still, 5 of the 35 subjects with ATS of less than 3.5 had CDT ≥ 1.6%. So, some false negatives for the ATS were heavy drinkers not identified by the ATS.

**Figure 4:**
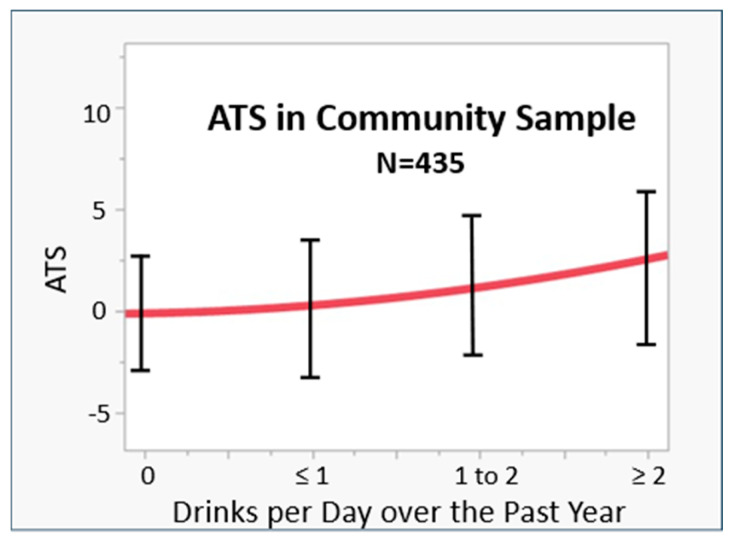
The distribution of ATS values in 435 community subjects characterized for alcohol intake. The 95% CI is indicated by error bars.

The above data show that the ATS is a powerful predictor of chronic HAC. But what about lower levels of consumption? Can the ATS be used for characterizing lower levels of alcohol consumption? To answer that question, we examined an alcohol dose–response curve using data from a community sample of subjects (*n* = 535; see Figure 4). As the figure shows, the average ATS value was zero for those who do not drink, with a steady non-linear increase in ATS as the average daily drinking consumption increased.

The relationship of the ATS to other clinical phenomena associated with HAC has been explored in a number of communications. The ATS is strongly associated with epigenetic aging [12,74,75]. The ATS strongly predicts immune WBC count [12]. Finally, and perhaps critically, for those interested in understanding the co-morbidity of smoking with drinking, the ATS has been repeatedly shown to strongly correlate (r values between 0.45 and 0.7) with cg05575921-indicated smoking intensity [12,61,69,74,75,76]. As discussed below, the ATS also predicts alcohol withdrawal seizures [61].

In summary, the ATS appears to be a reliable tool for predicting chronic HAC and clinical phenomena associated with chronic HAC. The non-linear dose responsiveness of the index for lower levels of consumption also suggests a potential for the use of the metric for studies attempting to quantify the health impact of lower levels of alcohol consumption.

## 7. A Potential Marker of Recent HAC

A shortcoming of the ATS is its inability to distinguish more recent (e.g., the past three weeks) from more remote HAC. This shortcoming is a natural consequence of the decision to select for markers predicting six or more weeks of HAC. This insensitivity to more recent HAC may be important because studies of prisoners conducted in the early 1950s showed that vulnerability to AWS can occur in as little as 30 days of HAC [77,78]. Hence, the ATS may not be positioned to be a strong predictor of AWS.

In our studies of smoking-induced DNA methylation, we noted that the loci that demethylated the fastest in response to the initiation of smoking were also the fastest to re-methylate in response to smoking cessation [63,79]. Therefore, in the hopes of isolating similar loci for alcohol, we analyzed genome-wide methylation data from two studies that collected DNA from subjects as they entered and exited 30-day alcohol treatment programs to identify those loci that not only predicted HAC but also had large changes in DNA methylation between the entry and exit time points [61]. These studies identified several candidate loci, but the one that showed the most reliable changes between studies was cg07375256, a residue in ZSCAN25, a gene previously associated with hypertension. We then translated the array-based methylation measure into a MSdPCR assay, then re-examined DNA samples from subjects entering and exiting alcohol treatment (an average time period of 23 days). During this ~three-week period, according to array-based assessments, the average methylation at this locus reverted over 5%, while its individual receiver operator characteristic area under the curve for HAC by itself was greater than 0.8.

**Figure 5:**
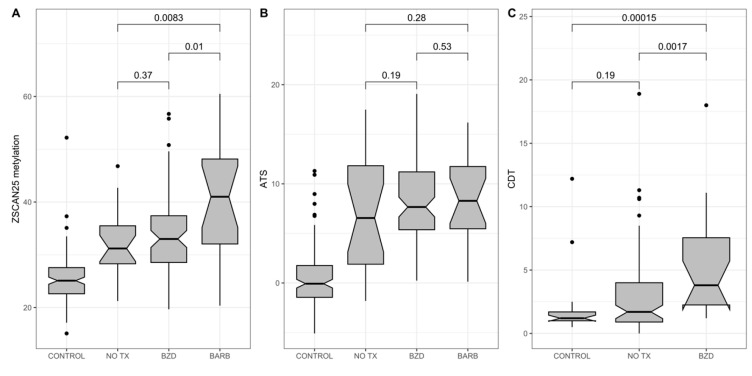
The relationship of (**A**) ZSCAN25 (%), (**B**) ATS (unitless), and (**C**) CDT (%) values with either control status (control) or alcohol withdrawal treatment status in a group of 125 subjects admitted for possible alcohol withdrawal syndrome (total *n* = 125). No tx (*n* = 32) signifies that no pharmaceutical treatment was administered. The benzodiazepine group (BZD) received only benzodiazepines (*n* = 74), while the barbiturate group (BARB, *n* = 19) received phenobarbital as an alcohol withdrawal preventative therapy. Data are from Andersen et al., 2023 [61].

Next, the array-based measurements were translated into a MSdPCR format, and we tested the hypothesis that cg07375256 (a.k.a. ZSCAN25) methylation would be a potent predictor of AWS and AWS-related phenomena in 120 subjects admitted for the consideration of AWS. Figure 5 shows the relationship between ZSCAN25 status and the ATS and the need for treatment with phenobarbital, which is commonly given by clinicians to patients thought to be at high risk for AWS [80], in 120 subjects admitted for consideration of AWS [61]. As Figure 5 shows, ZSCAN25 and CDT (which like the ATS is more sensitive to recent HAC) but not the ATS predicted the clinician’s choice to use phenobarbital.

**Figure 6:**
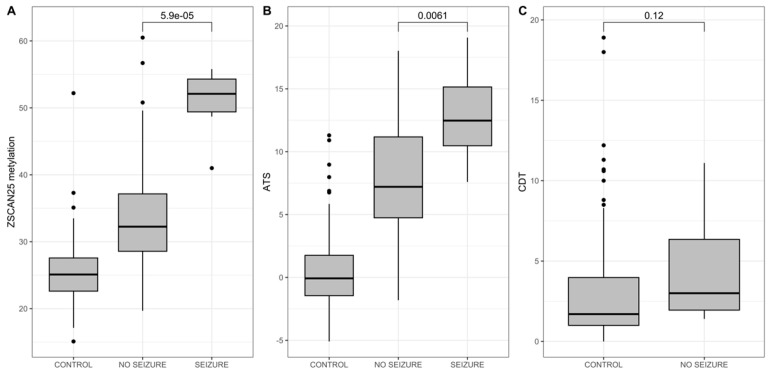
The relationship between (**A**) ZSCAN25 (Dcg07375256), (**B**) ATS, and (**C**) CDT and levels and presence (1) or absence (0) of seizures. N = 7 for the seizure group, 116 for the no seizure group. ZSCAN25 methylation and CDT values are given in percent. Receiver operating characteristic area under the curve (AUC) for predicting seizures is 0.95. Data are from Andersen et al., 2023 [61].

However, use of phenobarbital is an imperfect outcome at best since the criteria for using phenobarbital vary from practitioner to practitioner and not all clinicians at our institution can or will use phenobarbital for prevention of severe AWS. However, the occurrence of seizures is well recognized by all clinicians and is an easily assessed outcome associated with severe AWS. Therefore, the relationship of AWS-associated seizures with respect to the ATS, ZSCAN25, and CDT status was also analyzed. As Figure 6 demonstrates, ZSCAN25 and, to a lesser extent, the ATS, but not the CDT, were strong predictors of AWS-associated seizures.

Taken as a whole, these findings suggest that ZSCAN25 methylation has promise as a marker of relatively short-term HAC- and AWS-related phenomena. However, the current conclusions are based on data from only 120, largely White, midwestern AWS subjects. A more exacting understanding will require more extensive replication and extension.

## 8. A Pathway Forward

**Figure 7:**
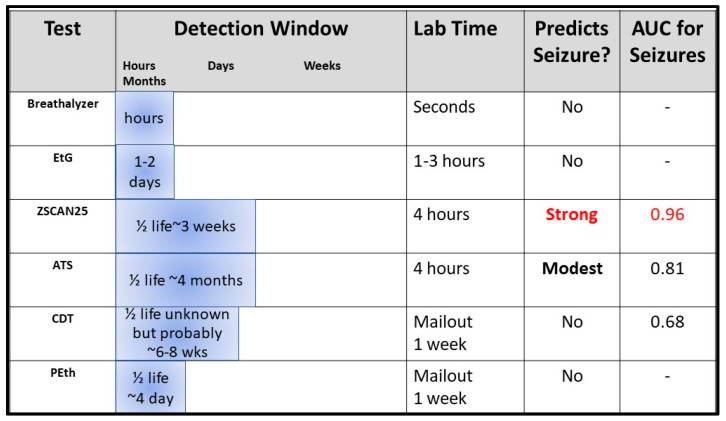
A comparison of the detection windows of the various testing technologies for alcohol consumption. The ZSCAN25 assay predicts HAC over a short term, where the window for HAC predicted by the alcohol signature is more remote. Please note that to date, none of the other tests have published evidence supporting their use in alcohol withdrawal. AUC values are from Andersen et al., 2023 [61].

Ultimately, the goal of any clinical biomarker is to advance the diagnosis, treatment, and prevention of illness. In fact, under the aegis of National Institutes of Alcohol and Alcohol Abuse (NIAAA) funding, the ATS and the ZSCAN25 markers were developed for exactly those purposes. Still, at best, they are only good additions to an existing armamentarium of tools for assessing patterns of alcohol consumption. Figure 7 outlines some key parameters with respect to the existing biomarkers of alcohol consumption. All of these biomarkers have significant value. However, depending on the question being asked, some biomarkers may be better suited than others.

Based on the available data, we believe that the ATS is well positioned as a predictor of chronic HAC, which we define as drinking an average of six or more drinks per day for eight or more weeks. Because the test can be performed quickly and easily by laboratories with digital PCR machines, is compatible with standard DNA-based NextGen testing methods, and is not subject to many of the limitations confronting liver-based assessments, we believe that it will increasingly become a more common method of assessing levels of sustained alcohol consumption for patients who would benefit from an objective assessment of potential HAC in both the psychiatric and non-psychiatric settings. For example, based on our work with those admitted for acute coronary syndrome [81], it appears that a substantial reduction in morbidity and mortality could be achieved by implementing evidence-based alcohol treatment therapies in many of those admitted for myocardial infarctions.

In addition, because of the relatively low cost of the test and minimal requirement for blood, requiring only a few hundred nanograms of DNA, the ATS has the potential to be used to retrospectively identify heavy alcohol consumption in biorepositories. This would, for example, be advantageous for addressing the role of alcohol in cancer. Alcohol is thought to be a potential risk factor for several types of cancer [82,83], but our current inability to quantify chronic consumption at scale precludes policymakers from being more certain.

In contrast, the exact utility of the ZSCAN25 marker has not yet been established. Certainly, the unique ability of this marker to strongly predict the occurrence of AWS-related seizures suggests the possibility that MSdPCR assessments could be used as part of the triage process for deciding which patients in the emergency room need to be admitted for potential serious AWS and which should be immediately referred to less cost-intensive alcohol treatment settings. Still, the hallmark of a robust biomarker is its ability to repeatedly predict in diverse populations. Furthermore, it may well be that like the ATS, it may be more advantageous to measure several sites rather than just one site, to gain predictive stability. Therefore, it is likely that many more clinical studies using additional markers and including patients from diverse settings will be needed before firm conclusions can be made about this approach for assessing the risk of severe AWS-related phenomena.

It is also possible that there are additional potential uses of DNA methylation to better characterize alcohol use disorders. For example, if we can identify methylation sites that hyper/hypomethylate in response to alcohol in a matter of days, they could serve as sentinels of very recent HAC. Likewise, we may gain a better understanding of the relationship of bursts of alcohol use, such as those seen in binge drinking, to changes in DNA methylation status, opening a range of important questions to more rigorous examination. But, given the nature of binge drinking, it is notoriously hard to collect uniform sets of subjects for case and control analyses. Finally, it may be possible isolate markers of remote, but not recent HAC. If so, these markers could fill a much-needed gap in helping understand the relationship between early HAC and later onset of illnesses, such as dementia.

No matter what the application, methylation-based biomarkers are tools whose utility relies on the right question being asked. For many questions, such as for determining alcohol intoxication or use in the past several days, it may be the wrong tool. Indeed, each of the assays listed in Figure 7 have significant utility under the right circumstances. Determining which of the options from Figure 7 is the best tool for detecting EAC may also require additional clinical information, such as the presence of co-morbidities or the use of certain medications. Finally, the best choice under certain circumstances may be to use several different biomarkers.

The first barrier to implementation of this technology is the need for access to digital PCR machinery. Currently, there are at least nine manufacturers of digital PCR machines, with the total market for 2023 for dPCR sales and services being estimated at USD 8.6 billion [84]. The exact number of machines that have been placed to date by each manufacturer is not known and appears to be a trade secret. But, given the rapid growth projections for this market [84], it is likely that most major diagnostic markets will have ready access to this technology in the near future.

However, the widespread adoption of these or any other markers is also hindered by the need for regulatory approval and reimbursement. Notably, none of the biomarkers currently used have received regulatory approval from the United States Food and Drug Administration or the European Medicines Agency. However, there are able to be offered as less well-regulated laboratory-developed tests (LDT) in the United States, with several of the methods having both Current Procedural Terminology (CPT) codes and some coverage by payors. Neither the ZSCAN25 or ATS have advanced to that point, and there are significant financial and logistical challenges to their advancement. However, we do note that the MSdPCR test for smoking is scheduled to be available as an LDT offered through Quantigen (Indianapolis, IN USA) in March 2025 and is currently undergoing pre-submission consideration by the FDA.

Finally, despite their clear promise, neither of these tests have been examined in large, diverse populations. Typically, before a test receives FDA approval, repeated analyses of test performance characteristics (i.e., sensitivity, specificity, and positive and negative prediction values) in large, well-characterized, diverse subjects must be performed. To date, these studies have yet to be conducted, and they will be necessary for optimal use of this test in clinical settings.

## 9. Conclusions

In summary, in this manuscript, we have detailed the development and performance characteristics of two MSdPCR tests for HAC. We believe that the rapid, precise, and affordable nature of MSdPCR makes these types of HAC assessments an option for both clinical and research applications, and that epigenetic tools will be increasingly utilized to guide care of AUD and allow researchers to more rigorously test key theoretical propositions regarding etiology, remission, and consequences of AUD.

## Figures and Tables

**Table 1:** Comparison of potential clinical methylation assessment methods.

Assay	Precision	Type	Reference	Speed	Cost
Array	Moderate	Global	Dependent	3–5 days	High
Sequencing	Variable	Variable	Independent	2–3 days	High
MSqPCR	Moderate	Single locus	Dependent	~4 h	low
MSdPCR	High	Single locus	Independent	~4 h	low

**Table 2:** A list of the CpG sites in the ATS.

Locus	Gene	Chromosome	Gene Function
cg02583484	HNRNPA1	12q13	RNA-binding protein
cg04987734	CDC42BPB	14q32	Kinase that regulates cytoskeleton and cell migration
cg09935388	GFI1	1p22	Zinc finger transcriptional repressor
cg04583842	BANP	16q13	Negative regulator of P53

## Data Availability

The datasets used to produce these graphics are available from the corresponding author upon reasonable request.
